# Editorial: Be positive about the negative in pharmacology: Ethnopharmacology 2022

**DOI:** 10.3389/fphar.2023.1259934

**Published:** 2023-08-15

**Authors:** Yusof Kamisah, Lie-Fen Shyur

**Affiliations:** ^1^ Department of Pharmacology, Faculty of Medicine, Universiti Kebangaan Malaysia, Kuala Lumpur, Malaysia; ^2^ Agricultural Biotechnology Research Center, Academia Sinica, Taipei, Taiwan

**Keywords:** traditional medicine, pharmacology, traditional Chinese medicine, toxicity, side effects

## 1 Introduction

Natural resources including plants provide a rich supply of new drugs (Newman and Cragg, 2020). Many plants have been used in folk medicine to alleviate the symptoms of various illness. Ethnopharmacology concerns scientific studies dealing with pharmacological properties of medicinal plants that are used by different ethnics (Taylor and Werneke, 2018). More often than anticipated, studies dealing with plants and their bioactive compounds are met with negative findings that nullify hypotheses, and this could be quite upsetting. It is a known fact that conducting a research is costly and time-consuming. Thus, repeating experiments with negative findings would be ultimately a waste of resources and time, which can involve millions of dollars lost.

The Research Topic *“Be positive about the negative in pharmacology: Ethnopharmacology 2022”* aims to collate articles that report negative and inconclusive findings in ethnopharmacology. It is a Research Topic of four articles focusing on adverse findings of medicinal plants. The publication of the Research Topic is to prevent duplications of experiments that produced negative results. Featuring negative results can advance science and allow other researchers to learn from them. It could also help to cater alternative approaches to tackle deviations from the prediction in science.

Diabetes mellitus is a common disorder that affects endocrine system and has become one of primary public health Research Topic. Many plants including *Parkia speciosa* Hassk. (Gao et al., 2023), *Syzygium polyanthum* (Wight.) Walp (Widyawati et al., 2022), and *Andrographis paniculata* (Burm.f.) Nees (Thakur et al., 2016). demonstrated hypoglycemic effects in diabetic rats. A randomized controlled trial was conducted to investigate the effects of *A. paniculata* and *S. polyanthum* mixture in addition to metformin therapy in 54 patients with type 2 diabetes mellitus. A reduction in fasting blood glucose and postprandial glucose was seen in the patients receiving the treatment (Widjajakusuma et al., 2019).

Despite many positive reports of *A. paniculata* effects on blood glucose level in rodents, Suemanotham et al. reported that the plant extract did not exhibit any beneficial effects on fasting blood sugar, inflammatory, and oxidative stress biomarkers in diabetic canines. A possible explanation for the discrepancy is interspecies variation in response. Studies on the plant antidiabetic property in rodents adopted streptozotocin- or alloxan-induced type 2 diabetes mellitus model (Thakur et al., 2016; Wediasari et al., 2020), while Suemanotham et al. study used dogs which suffered from type 1 diabetes mellitus due to autoimmune damage to *β*-cells of the pancreas, the most prevalent type of canine diabetes. Therefore, it is possibly that *A. paniculata* is only effective against type 2 diabetes mellitus, but not against the type 1.

Liver being the primary organ for xenobiotic metabolism, is a common target for drug-induced toxicity. Hepatotoxicity is one of the causes for the withdrawal of many therapeutic drugs from market (Mirahmad et al., 2022). Liver toxicity is commonly accompanied by inflammation and oxidative damage. Certain traditional Chinese medicines have been reported to manifest hepatotoxicity. Wang et al. described the hepatotoxic effects of dried roots of *Polygonum multiflorum* Thunb. (*Polygoni Multiflori Radix*) and *Rheum palmatum* L. (*Rhei radix et rhizoma*) in mice. It is believed that the toxic effects are due to the presence of tetrahydroxystilbene glucoside and emodin glucoside in the roots, which correspond to their relatively higher concentrations in the liver. *Polygoni Multiflori Radix* contains both compounds, while *Rhei radix et rhizome* only contains the latter. Reduced hepatic antioxidant capacity and elevated hepatic inflammation were also observed in the groups receiving the medications.

Danlu tongdu tablet is another traditional Chinese medicine which exerts hepatotoxicity in rats (Zhang et al.). It comprises a mixture of *Astragalus membranaceus* (Fisch.) Bge (*Astragali radix*), glue of deer antler (*Cervi cornus Colla*)*, Eucommia ulmoides* Oliver (*Eucommiae cortex*), *Salvia miltiorrhiza* Bunge (*Salviae miltiorrhizae radix et rhizoma*), and *Corydalis yanhusuo* W.T.Wang (*Corydalis rhizoma*). Oral administration of the tablet at a high dose (6.67 g/kg body weight) up to 26 weeks caused mild hepatic hyperplasia and hypertrophy which were reversible. The medication was noted to upregulate the expression of cytochrome P4501A—CYP1A1 and CYP1A2—a mechanism which might account for the toxicity. Induction of both isoenzymes is associated with hepatic inflammation and oxidative damage (Hussain et al., 2014).

Triptolide is a primary bioactive compound in *Tripterygium wilfordii* Hook., an herb commonly used in traditional Chinese medicine. Li et al. found the compound increased liver inflammation and lipid accumulation in zebrafish. However, in the presence of inflammatory state induced by lipopolysaccharides, the detrimental effects of the compound were worsened. The injurious effects of triptolide were likely associated with impaired regulation of genes involved in lipid metabolism, oxidative stress, apoptosis, and autophagy. Therefore, it may pose a concern regarding its administration in patients with existing inflammatory condition.

In conclusion, the Research Topic presents updated negative research findings in ethnopharmacology with more understanding on mechanistic aspects. The use of medicinal plants are not without adverse effects, and some may be ineffective. More studies are needed to provide more comprehension of the negative effects. While publication of inconclusive findings would prevent further repetition, hence minimizing the risk of squandering invaluable resources.
